# Antiepileptic drug adherence in children in southern Ethiopia: A cross sectional study

**DOI:** 10.1371/journal.pone.0263821

**Published:** 2022-02-17

**Authors:** Shamil Ahmed Dima, Mulugeta Sitot Shibeshi

**Affiliations:** 1 Department of Pediatrics and Child Health, Madda Walabu University, Gobba, Ethiopia; 2 Department of Pediatrics and Child Health, Hawassa University, Hawassa, Ethiopia; Cook Children\’s Health Care System: Cook Children’s Medical Center, UNITED STATES

## Abstract

**Background:**

Epilepsy is one of the commonest chronic neurological disorders with serious health consequences. Treatment adherence is one of the determinants of seizure control. This study was designed to determine factors affecting antiepileptic drug adherence among children with epilepsy.

**Methods:**

A cross sectional study was conducted on 192 children with epilepsy (≤14 years of age) on follow-up at a pediatric neurology clinic in Southern Ethiopia from January 1^st^ to August 30^th^, 2019. Medication Adherence was measured using the eight-item Morisky’s medication adherence scale. Logistic regression analysis was done to determine factors associated with antiepileptic drug adherence.

**Result:**

One hundred twenty-five (65%) of the study subjects were adherent to their medication. On multivariable analysis, factors predictive of good adherence included family size of ≤5 [AOR = 2.34, (95% CI: 1.07, 5.10); P = 0.03] and duration of epilepsy (<1year [AOR = 5.83, (95% CI: 1.48, 22.92); P = 0.012] and 1-2year [AOR = 4.58, (95% CI: 1.12, 18.77); P = 0.035]). Monthly family income of <1000 Ethiopian Birr [AOR = 0.18, (95% CI: 0.06, 0.61); P = 0.005] and presence of seizure attack in the past 3months [AOR = 0.23, 95% (CI: 0.10, 0.55); P = 0.001] predicted poor antiepileptic drug adherence.

**Conclusion:**

Adherence to antiepileptic drugs in children is low in our setting; low family income and occurrence of seizures while on treatment predicted poor adherence. Supplying free antiepileptic drugs to poor children and regular provision of information about expected treatment response to children with epilepsy and their caretakers may help improve adherence.

## Introduction

Epilepsy is a disorder of the brain characterized by an enduring predisposition to generate epileptic seizures and by the neurobiological, psycho logical, and social consequences of this condition [1].

Prevalence studies of epilepsy in Sub-Saharan Africa varied from 2 per 1000 to 134.5 per 1000 persons [2]. The wider variation in the estimates of prevalence is related to differences in the distribution and types of risk factors for epilepsy [3] and the methodology used [2]

The burden of epilepsy in Ethiopia is not well documented, though the estimated prevalence is 1.4% [4]. A community-based study in Ethiopia revealed an annual incidence of 64 in 100,000 inhabitants, the highest incidence being in children aged 0–9 years [4]. On the other hand, a very high prevalence rate of epilepsy (29.5/1000) was found in the Zay Society of Ethiopia, an endogamous community of approximately 1000 people [5].

The goal of treatment of epilepsy is to maintain a normal lifestyle, free of seizures and with minimal side-effects while on medication. Previous studies showed that 60–80% of patients with epilepsy had well controlled seizures with antiepileptic drugs (AEDs), but for this to be achieved adherence to medication should be observed [6–8].

Adherence refers to how patient treatment related behaviors correspond to health professionals’ advice [9]. It needs a good patient involvement in treatment as well as a mutual arrangement of cooperation and agreement between the health provider and the patient [8]. Poor adherence to prescribed medication is considered to be the main cause of unsuccessful drug treatment for epilepsy [7,8]. Non-adherence is associated with increased morbidity and mortality, reduced quality of life, and increased health care costs [9–11].

Measuring adherence has considerable methodological problems; and no single measurement strategy has been deemed optimal [6]. For example, measurement of serum levels of anticonvulsant drugs, which is believed to be the best indicator of adherence, has its own limitations [12]. It may be influenced by factors other than adherence, including altered pharmacokinetics resulting from co-medication, poor absorption, and genetic differences in drug metabolic rates [13]. Self-report, which has been the main method of assessing adherence in low resource settings, is reported to overestimate adherence [14,15]. Although prone to social desirability and recall biases [16], self-report measures show moderate correspondence to other adherence measures and can provide actionable information [15].

Estimates of adherence to antiepileptic drugs in children are variable in different studies depending on the population studied and method used. A study in Uganda revealed that AED adherence by self-report was 80% but only 22% by drug levels [14]. On the other hand, a multiple-methods assessment of adherence to AEDs revealed non-adherence in 33% of children with epilepsy in Northern Ireland [17].

Adherence is influenced by several factors that include socioeconomic factors, the health care system, the characteristics of the disease, the treatment the patient receives, and patient-related factors [16]. Some of the predictors of adherence identified in previous studies include duration of treatment [8,18,19], seizure type and parental depressed mood [17], seizure frequency [20], pill burden and drug costs [16], comorbidities [10,21–23], stigma [20,22], parent and child satisfaction with medical care [24], and medication adverse effects [21,22,25]. There is scarcity of data on treatment adherence and associated factors among children with epilepsy in Ethiopia. This study assessed the status of adherence to antiepileptic drugs and identified some of its predictors in children with epilepsy in southern Ethiopia.

## Methods and materials

### Study area

The study was conducted at the pediatric neurology referral clinic of Hawassa University Comprehensive Specialized Hospital located about 275 km south of Addis Ababa, the capital city of Ethiopia. The hospital provides neurodiagnostic services (electroencephalography (EEG), CT scan and MRI). The pediatric neurology follow-up clinic operates once weekly, and on average 15 to 20 children are evaluated on each follow-up day. The clinic is attended by a pediatric neurologist, 2–3 pediatric residents, and a nurse to evaluate patients on regular follow-up. During each visit, patients are evaluated for seizure control, AED treatment adherence, AED adverse effects, growth and development, and are provided with education relevant to their disease.

### Study design, subjects and sample

The study was a hospital based cross-sectional study among children with epilepsy ≤ 14 years of age from January 1^st^ to August 30^th^, 2019.

We calculated sample size using a single population proportion formula with the following assumptions: a 50% prevalence of adherence in children with epilepsy as no similar research has been conducted in Ethiopian children, a 5% precision, and a 95% level of confidence, yielding a sample size of 384. Since the overall number of children with epilepsy in our hospital was less than 10,000 (i.e. 192), a correctional formula (nf = ni×N/ni+N) was utilized. Where, nf = final sample size, ni = sample size from single population proportion formula, N = number of children with epilepsy in our hospital, and substituting all the variables resulted in nf = 384×192 /384+192 = 128. To compensate for the non-respondents, 10% of the calculated sample size was added bringing the total sample size to 141. However, since the number of children with epilepsy who had follow-up in our hospital was small and manageable, we recruited all children with epilepsy who were willing to give informed consent when they came for their regular follow-up.

### Data collection procedure

Data were collected using a pretested questionnaire that contained questions about sociodemographic and epilepsy related information. Sociodemographic information includes age, sex, and address of the child. Epilepsy-related information includes age at diagnosis, epilepsy type, syndrome status, comorbid neurological conditions, prescribed antiepileptic drugs, medication adherence profile, patient’s access to AEDs, and knowledge about epilepsy. Caretakers’ knowledge about epilepsy was assessed using a five item tool consisting of questions about the cause, treatment and prognosis of epilepsy. As a significant percentage (40.1%) of the caretakers had low educational level (12.5% were illiterate and 27.6% had only elementary education), we took the mean score (≥50%) to classify caretakers as knowledgeable or unknowledgeable. Hence, care takers who scored more than and equal to the mean score were considered knowledgeable and those with a score of less than the mean unknowledgeable.

Patients (age ≥10years) and caretakers were interviewed, patients’ medical records were reviewed and information pertinent to their epilepsy was recorded on the data collection tool by trained nurses when they came for their follow-up visits.

Antiepileptic drug adherence was measured by an adopted version of the eight-item Morisky Medication Adherence Scale (MMAS-8) [26–28]… It is among the most widely used tools to assess non-adherence to medications and has been validated in diverse population groups [29]. The total score of MMAS-8 was eight. A higher score indicates a higher level of self-reported adherence. Accordingly, patients who had an MMAS-8 score of 8 were considered to be adherent and those with a score of less than 8 non-adherent.

### Data processing and analysis

Data were entered in the Statistical Package for Social Sciences software (version 23) for windows after cleaning, and descriptive and analytic statistics were done as applicable. Patients’ sociodemographic characteristics and epilepsy related variables were summarized using frequency distribution tables. Mean/median and standard deviation/inter quartile range were calculated for continuous data. Bi-variate logistic regression analysis was carried out to determine candidate variables (with a p-value < 0.05) for the multivariable logistic regression model. A P-value of < 0.05 was considered statistically significant.

### Ethical consideration

The study was conducted after obtaining ethical clearance from the Institutional Review Board of Hawassa University, College of Medicine and Health Sciences (IRB No.: IRB/071/11). Written informed consent from parents/guardians and assent from adolescents was obtained. Anonymity was maintained throughout the study period. After determination of the status of antiepileptic drug adherence of the study subjects, patients/caretakers were counseled on measures to improve adherence. Participants had the right not to participate or withdraw from the study at any point.

## Results

A total of 192 children with epilepsy (66.7% males), aged 3 months to 14 years participated in the study. The mean age of the participants was 6.85 ± 4.24 years. Socio-demographic characteristics of participating children and their caregivers are shown in Table 1.

**Table 1:** Socio-demographic characteristics of caregivers and children on AEDs (n = 192).

Variables	Category	Frequency	Percent
Child’s sex	Male	128	66.7
	Female	64	33.3
Child’s age	3months-2yr	24	12.5
	2-6yr	53	27.6
	6-10yr	47	24.5
	10-14yr	68	35.4
Educational status of the child (if >4yrs of age)	Did not join school	20	17.4
	Attending school	79	68.7
	Dropped out of school	16	13.9
Family size	≤ 5	109	56.8
	>5	83	43.2
Primary Caregivers’ age	15–24	9	4.7
	25–34	68	35.4
	35–44	88	45.8
	≥ 45	27	14.1
Primary Caregivers’ sex	Male	88	45.8
	Female	104	54.2
Place of residence	Urban	148	77.1
	Rural	44	22.9
Caregivers’ religion	Protestant Christian	76	39.6
	Orthodox Christian	66	34.4
	Muslim	41	21.4
	Others	9	4.7
Caregivers’ educational level	Can’t read or write	24	12.5
	Elementary	53	27.6
	High School	49	25.5
	Diploma	40	20.8
	Degree and above	26	13.5
Caregivers’ occupation	Farmer	43	22.4
	Merchant	52	27.1
	Government employee	62	32.3
	NGO employee	22	11.5
	Jobless	13	6.8
Caregivers’ relation to child	Biological parents	174	90.6
	Grand parents	6	3.1
	Volunteers	6	3.1
	Others	6	3.1
Average monthly income of the family	<1000_ETB	32	16.7
	1000–2000_ETB	41	21.4
	2000–3000_ETB	32	16.7
	≥3000_ETB	87	45.3

ETB: Ethiopian Birr; 1USD = 29.1 ETB during the study time.

Epilepsy was generalized in 156 children (81.3%), focal in 29 (15.1%) and unclassified in the rest. Only 105 (54.7%) of the study subjects had EEG recording, and 55 of them (52.4%) had abnormal EEG finding. Focal epileptiform discharge was the most common EEG abnormality (50.91%). Twenty nine patients (15%) had family history of epilepsy. Of all study subjects, 32 (16.7%) had comorbid conditions. Cerebral palsy was the most common comorbidity (46.9%). Clinical characteristics of participating children are shown in Table 2.

**Table 2:** Frequency distribution of AED adherence related characteristics (n = 192).

Variables	Category	Frequency	Percent
**Access to Antiepileptic Drugs**			
Distance from neurology clinic	<10km	53	27.6
	10-20km	27	14.1
	20-30km	29	15.1
	30-40km	9	4.7
	≥40	74	38.5
Frequency of appointments	Weekly	3	1.6
	Every 2 weeks	10	5.2
	Monthly	110	57.3
	Every 2 months	53	27.6
	Every 3 months	16	8.3
Source of AEDs	With payment	184	95.8
	Free	8	4.2
Financial problem	Yes	76	39.6
	No	116	60.4
Availability of prescribed drugs all the time	Yes	131	68.2
	No	61	31.8
**Clinical factors**			
Seizure type	Generalized	156	81.3
	Focal	29	15.1
	Unclassified	7	3.6
Electroencephalographic features(n = 105)	Focal epileptiform discharge	28	26.7
	Focal to bilateral	3	2.9
	Generalized epileptiform discharge	24	22.8
	Normal	50	47.6
Duration of illness	<1yr	64	33.3
	1-2yr	46	24.0
	2-3yr	25	13.0
	≥3yr	57	29.7
Seizure attack in the past 3months	Yes	107	55.7
	No	85	44.3
Duration of treatment	≤2yr	135	70.3
	>2yr	57	29.7
Number of AEDs the child was taking	Monotherapy	145	75.5
	Polytherapy	47	24.5
Drug formulation	Tablets	175	91.1
	Suspension/syrup	12	6.3
	Both tablet and suspension/syrup	5	2.6
Frequency of daily doses	Once	11	5.7
	Twice	175	91.1
	Three times	6	3.1
Type of comorbidity (n = 32)	Cerebral palsy	15	46.9
	Developmental delay	8	25
	ADHD	4	12.5
	Down syndrome	3	9.4
	Autism Spectrum Disorder	2	6.2
**Caregivers’ knowledge (about epilepsy) and substance use status**			
Knowledge of caregivers about epilepsy and its treatment	Unknowledgeable	93	48.4
	Knowledgeable	99	51.6
History of substance use	Yes	6	3.1
	No	186	96.9
Type of substance used	Khat	2	33.3
	Cigarette	2	33.3
	Alcohol	2	33.3
Stigmatized because of epilepsy	Yes	30	15.6
	No	162	84.4

**Fig 1:**
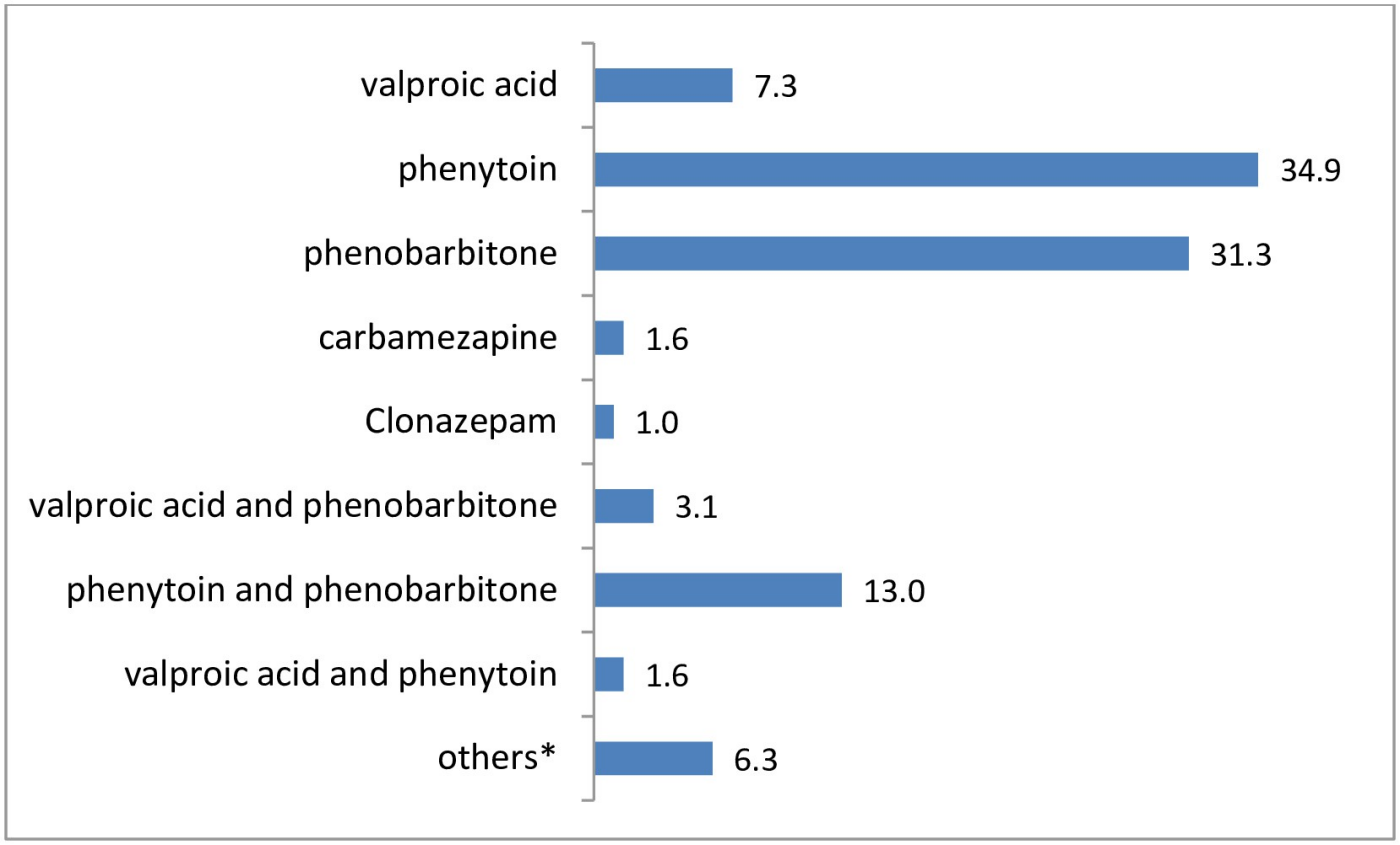
Percentage distribution of commonly prescribed AEDs. * Valproic acid and clonazepam (n = 2); phenobarbitone and carbamazepine (n = 2); lamotrigine(n = 2); Valproic acid, phenobarbitone and clonazepam(n = 2); phenytoin and carbamazepine(n = 2), Valproic acid, carbamazepine and clonazepam(n = 1).

One hundred seventy-eight (92.7%) of the patients/caregivers had received counseling before they started their treatment. Caretakers always administered and/or supervised administration of the AEDs in the adolescent patients. Majority (75.5%) of patients were on monotherapy, and phenytoin was the most commonly prescribed drug (34.9%) (Fig 1).

Based on the eight-item Morisky’s medication adherence scale, 125 (65%) patients were adherent to their treatment. Responses to AED adherence assessing items is given in Table 3.

**Table 3:** Responses to AED adherence assessing items.

Variables	Category	Frequency (N = 192)	Percent
Do you sometimes forget to take/give medicine?	No	149	77.6
	Yes	43	22.4
Within the last 2weeks, were there any days you did not take your medicine?	No	158	82.3
	Yes	34	17.7
Has the child ever stopped taking medicine without telling the doctor because he/she felt worse when he/she took it?	No	165	85.9
	Yes	27	14.1
When traveling or leaving home, do you sometimes forget medicine at home?	No	174	90.6
	Yes	18	9.4
Did the child miss taking any of his/her medicines yesterday?	No	187	97.4
	YES	5	2.6
Does the child sometimes stop taking his/her medicine when improved?	No	169	88.0
	Yes	23	12.0
Do you ever feel hassled about sticking to the treatment plan due to the inconvenience of taking drugs?	No	160	83.3
	Yes	32	16.7
Have you ever had difficulty remembering to give all the medicines to your child?	No	139	72.4
	Yes	53	27.6

From multivariable analysis, children from small families (n≤5) were 2.34 times more likely to be adherent to AEDs than those from a family size of >5 [AOR = 2.34, (95% CI: 1.07, 5.10); P = 0.03]. Children with an average monthly family income of <1000ETB were 82% less likely to be adherent to AEDs than those with an average monthly family income of ≥3000ETB [AOR = 0.18, (95% CI: 0.06, 0.61); P = 0.005]. Children with duration of epilepsy of < 1year were 5.83 times more likely to be adherent to AEDs than those with duration of epilepsy of ≥3years [AOR = 5.83, (95% CI: 1.48, 22.92); P = 0.012]. Similarly, children with duration of illness of 1-2years were 4.58 times more likely to be adherent to treatment than those with duration of illness of ≥3years [AOR = 4.58, (95% CI: 1.12, 18.77); P = 0.035]. Children who had seizure attacks in the past 3 months were 77% less likely to be adherent to AEDs than those who had no seizure attack [AOR = 0.23, (95% CI: 0.10, 0.55); P = 0.001]. (Table 4).

**Table 4:** Bi-variable and multivariable analysis of factors associated with AEDs adherence among children with epilepsy (N = 192).

Variable	Category	Frequency		COR (95% CI)	AOR (95% CI)
		Adherent (%)	Non-adherent (%)		
Family size	≤ 5	82(65.6)	27 (40.3)	2.83(1.53,5.21)*	2.34(1.07, 5.10)**
	>5	43(34.4)	40 (59.7)	1	
Average monthly family income(ETB)	<1000	11(8.8)	21(31.3)	0.17(0.07,0.40)*	0.18(0.06, 0.61)**
	1000–2000	25(20)	16(23.9)	0.50(0.22,1.10)	0.72(0.25, 2.10)
	2000–3000	23(18.4)	9(13.4)	0.81(0.33,2.03)	1.22(0.34, 3.79)
	≥3000	66(52.8)	21(31.3)	1	
Financial problem	Yes	36(28.8)	40(59.7)	0.27(0.15,0.51)*	0.70(0.29, 1.67)
	No	89(71.2)	27(40.3)	1	
Duration of illness	<1yr	52(41.6)	12(17.9)	4.49(1.99,10.14)*	5.83(1.48, 22.92)**
	1-2yr	34(27.2)	12(17.9)	2.94(1.27,6.79)*	4.58(1.12, 18.77)**
	2-3yr	11(8.8)	14(20.9)	0.814(0.32,2.09)	0.68(0.17, 2.75)
	≥3yr	28(22.4)	29(43.3)	1	
Seizure attack in the past 3 months	Yes	55(44.0)	52(77.6)	0.23(0.12,0.45)*	0.23(0.10, 0.55)**
	No	70(56.0)	15(22.4)	1	
Duration of treatment	≤2yr	97(77.6)	38(56.7)	2.64(1.39,5.02)*	0.74(0.22, 2.45)
	>2yr	28(22.4)	29(43.3)	1	
Number of AEDs the child was taking	Mono-therapy	101(80.8)	44(65.7)	2.20(1.12,4.31)*	0.000
	Poly-therapy	24(19.2)	23(34.3)	1	
Believe that devil causes epilepsy	No	42(33.6)	37(55.2)	0.41(0.22,0.75)*	0.47(0.21, 1.04)
	Yes	83(66.4)	30(44.8)	1	

*candidate variables for multivariable analysis at P-value< 0.05.

**significant at P-value <0.05; (1) reference category.

## Discussion

AED adherence in our setting was suboptimal, with only 65% of children adhering to their medications according to their self or parental reports. This finding indicates an area that needs improvement in the treatment of epilepsy, as poor adherence is one of the major causes of non-responsiveness to antiepileptic drug therapy [9,16].

The level of adherence documented in this study is similar to that reported from Iran [30] and Japan [31]. Similarly, our finding is in line with the result of a systematic review and meta-analysis from Ethiopia involving adult patients that revealed a pooled prevalence of AED non-adherence of 39.77% [21] However, the level of adherence noted in our study is lower than those reported in other studies [14,32]. Although there is a difference in the method used to measure adherence, our study showed a higher level of adherence than those reported by other authors [23,33,34]. The relatively higher adherence level in our setting, however, should be interpreted cautiously as self-reported adherence may not reflect the actual level of adherence due to the recall and social desirability biases associated with this method [16]. Self or parental reports frequently overestimate actual adherence. For instance, a study that utilized both self-report and serum drug levels revealed an adherence level of 80% by self-report but only 22% by drug levels [14].

The most common reasons for non-adherence were difficulty of remembering (27.6%) and forgetfulness (22.4%). Similar findings were reported in other studies [20,35,36]. In this setting, forgetfulness/ difficulty remembering can be address by medication adherence aids such as an automated medication reminder application for mobile phones [37,38] and involving additional family members in the care of children with epilepsy [38]. As caretakers with low literacy may have difficulty understanding instructions, less complex treatment regimens should be prescribed [38].

In this study, the presence of seizure in the past 3 months was associated with poor adherence. This could be probably because patients might have perceived negligible benefits from the AEDs when seizure recurred while they were adhering to the treatment. Similar findings were reported from Jordan and India [34]. Low family income adversely affected AED adherence in our study because most patients pay for AEDs, and some of the drugs are not readily available in public pharmacies. Hence, patients with low income would have poor adherence as they cannot afford to buy these drugs from the more expensive private pharmacies. This is in agreement with results of studies from Kenya [19] and Ethiopia [39]. However, other indicators of socioeconomic status such as caretaker’s education and occupation had no association with AED adherence in our study.

In this study, family size of ≤5 was identified as one of the factors associated with better drug adherence. This finding is in line with findings of studies from Iran [30] and India [34]. Children with epilepsy from smaller families would get better attention, and their parents are more likely to take part in epilepsy management activities (for example, regular administration or supervision of AED administration). Moreover, families with a small number of dependent children would have less financial pressure to access AEDs; and children from these families would have better treatment adherence than those from larger families.

The present study showed that duration of epilepsy was significantly associated with medication adherence. This finding is in agreement with results of studies from Ethiopia [22], Kenya [19] and Jordan [18]. This could be due to provision of inadequate information about the course of the illness and its possible prognosis at key points during the course of epilepsy treatment [40], the side effects of antiepileptic drugs taken for a prolonged period of time [21], or simply adherence fatigue from several years of drug administration [41]. However, a study from Iran did not show an association between duration of epilepsy and adherence to treatment [30].

Although stigma has been reported in previous studies as an important factor associated with treatment non adherence [20,22], this study has not demonstrated the association. Moreover, our study did not show an association between adherence and the presence of comorbidity in contrast to earlier findings [10,21–23]. Generalized seizures were the most common seizure type in this study (81.3%) which supports previous reports from Ethiopia that reported generalized convulsive seizures in 69%-81% of people with epilepsy [4,42]. However, EEG reports revealed that focal seizures were the most common EEG abnormality in this setting (50.91%) which is in line with another Ethiopian study that showed focal epileptiform discharges in 63.7% of patients [43]. Some of the subjects with clinically generalized seizures but having focal epileptiform discharges may have focal to bilateral tonic clonic seizures in which the initial focal phase of the seizures might have been missed by the caretakers. In this study, seizure type did not determine adherence. However, it was reported to be one of the determinants of adherence in a previous study that utilized multiple-methods(19). This study had some limitations. As some of the data used in the study were self-reported by patients and/or their caregivers, recall bias that could affect adherence levels might have occurred. Utilization of additional adherence measuring tools (serum AED concentration, electronic monitoring, and pill count) along with the self-report method might have revealed a different result [44]. We have not exhaustively studied the factors that were incriminated as determinants of adherence in other studies as some of the clinical data regarding our study subjects were incomplete. Moreover, as children in the community who might not have received modern treatment were not included in the study, our finding may not reflect the true level of adherence in children with epilepsy in Southern Ethiopia.

In conclusion, adherence to AED treatment in children is low in our setting. Appropriate interventions that address modifiable determinants of poor adherence are needed to improve AED compliance, and consequently treatment outcome. Some of the measures could be providing free AEDs to children with financial constraints and to those with large family size. Relevant information about the illness and its treatment should be provided during each visit to epileptic children and their caretakers to enhance treatment adherence. We recommend a large community based study by employing combinations of measures of adherence to better evaluate AED treatment adherence in children with epilepsy in our region.

## Supporting information

S1 Data (SAV)

## Abbreviations

AED: anti-epileptic drugs
AOR: adjusted odds ratio
CI: confidence interval
COR: crude odd ratio
CT scan: computed tomography
EEG: electroencephalogram
IRB: institutional review board
MMAS-8: 8 item Morisky medication adherence scale
MRI: magnetic resonance imaging
